# Phonology is not accessed earlier than orthography in Chinese written production: evidence for the orthography autonomy hypothesis

**DOI:** 10.3389/fpsyg.2015.00448

**Published:** 2015-04-17

**Authors:** Qingfang Zhang, Cheng Wang

**Affiliations:** ^1^Department of Psychology, Renmin University of ChinaBeijing, China; ^2^Key Laboratory of Behavioral Science, Institute of Psychology – Chinese Academy of SciencesBeijing, China

**Keywords:** written production, spoken production, orthography, phonology, orthographic autonomy hypothesis

## Abstract

The contribution of orthographic and phonological codes to written production remains controversial. We report results using a picture–word interference task in which participants were asked to write (Experiments 1 and 2) or to speak (Experiment 3) the names of pictures while trying to ignore visual distractors, and the interval between the target and distractor onset was varied. Distractors were orthographically plus phonologically related, orthographically related, phonologically related, or unrelated to picture names. For written production, we found an exclusive orthographic effect at an early stage, reflecting a fast and direct link between meaning and graphemic lexicon, and we demonstrated that orthographic codes can be accessed directly from meaning in healthy adults. We also found orthographic and phonological effects at a later stage, reflecting a slow and indirect link between meaning and graphemic lexicon via phonology. Furthermore, the absence of an interaction effect of orthographic and phonological facilitation on written latencies suggests that the two effects are additive in general and that they might occur independently in written production in Chinese. For spoken production, we found that orthographic and phonological effects occur simultaneously in spoken production and that the two effects are additive at an early stage but interactive at a later stage. The temporal courses and their interplay of orthographic and phonological effects are dissociative in written and spoken production. Our findings thus support the orthography autonomy hypothesis, rather than the obligatory phonological mediation hypothesis, in written production in Chinese (as a non-alphabetic script).

## Introduction

Over the past few decades, much research has investigated the processes and mechanisms underlying spoken production (Dell, 1986, 1988; Levelt et al., 1999). However, less work has been devoted to understanding written production. The current views of speech production provide a general theoretical framework from which hypotheses specific to writing can be derived (Bonin et al., 1997, 1998a,b, 2013; Rapp et al., 1997; Bonin and Fayol, 2000; Baus et al., 2013; Damian and Qu, 2013; Zhang and Wang, 2014). In the work reported here, we investigated how orthographic codes are accessed from the conceptual/semantic level in writing using a picture–word interference (PWI) paradigm, which is an experimental paradigm that is popular in speech production (i.e., Schriefers et al., 1990; Starreveld and La Heij, 1995, 1996a,b; Damian and Martin, 1999).

A central debate in the field is the contributions of orthographic and phonological codes (e.g., Qu et al., 2011; Bonin et al., 2013; Damian and Qu, 2013). Early theoretical accounts claimed that the retrieval of an orthographic representation was entirely dependent on the prior retrieval of phonological codes, which is called the *OBLIGATORY phonological mediation hypothesis* (Geschwind, 1969; Luria, 1970). This account is compatible with the common introspective speaking experience in writing (Hotopf, 1980) and the observation of phonologically mediated spelling errors such as homophone substitutions (e.g., *there* for *their*) or quasi-homophone substitutions (e.g., *dirth* for *dearth*; Aitchison and Todd, 1982). Evidence in brain-damaged patients and normal writers indicates that spelling errors occur more frequently with inconsistent rather than consistent spelling (i.e., Bonin et al., 2001), reflecting that phonology influences orthographic output. Neuropsychological patients with writing disorders present comparable impairments in spoken and written production (Luria, 1970; Basso et al., 1978), as the phonological mediation hypothesis predicts.

However, neuropsychological studies have demonstrated dissociations between spoken and written production. Studies have repeatedly reported that the ability to spell is often spared even when phonological production is severely damaged (e.g., Caramazza et al., 1983; Hanley and McDonnell, 1997). Miceli et al. (1997) reported a patient who, when presented with a picture, sometimes generated different spoken and written responses (e.g., in response to a picture of a *cook*, the patient would say *dish* but write *forks*; see Alario et al., 2003 for a similar case study). Rapp et al. (1997) presented the case of a neurologically impaired individual who was often able to write the names of pictures correctly but was unable to provide the correct spoken names of the pictures (e.g., in response to a picture *of tweezers*, the patient would say *pliers* but write *needle*; see also Caramazza, 1997 for details). These findings motivated the “*orthographic autonomy hypothesis,”* which assumes that individuals can gain access to orthographic representation directly from meaning without phonological mediation (Rapp and Caramazza, 1997).

**FIGURE 1:**
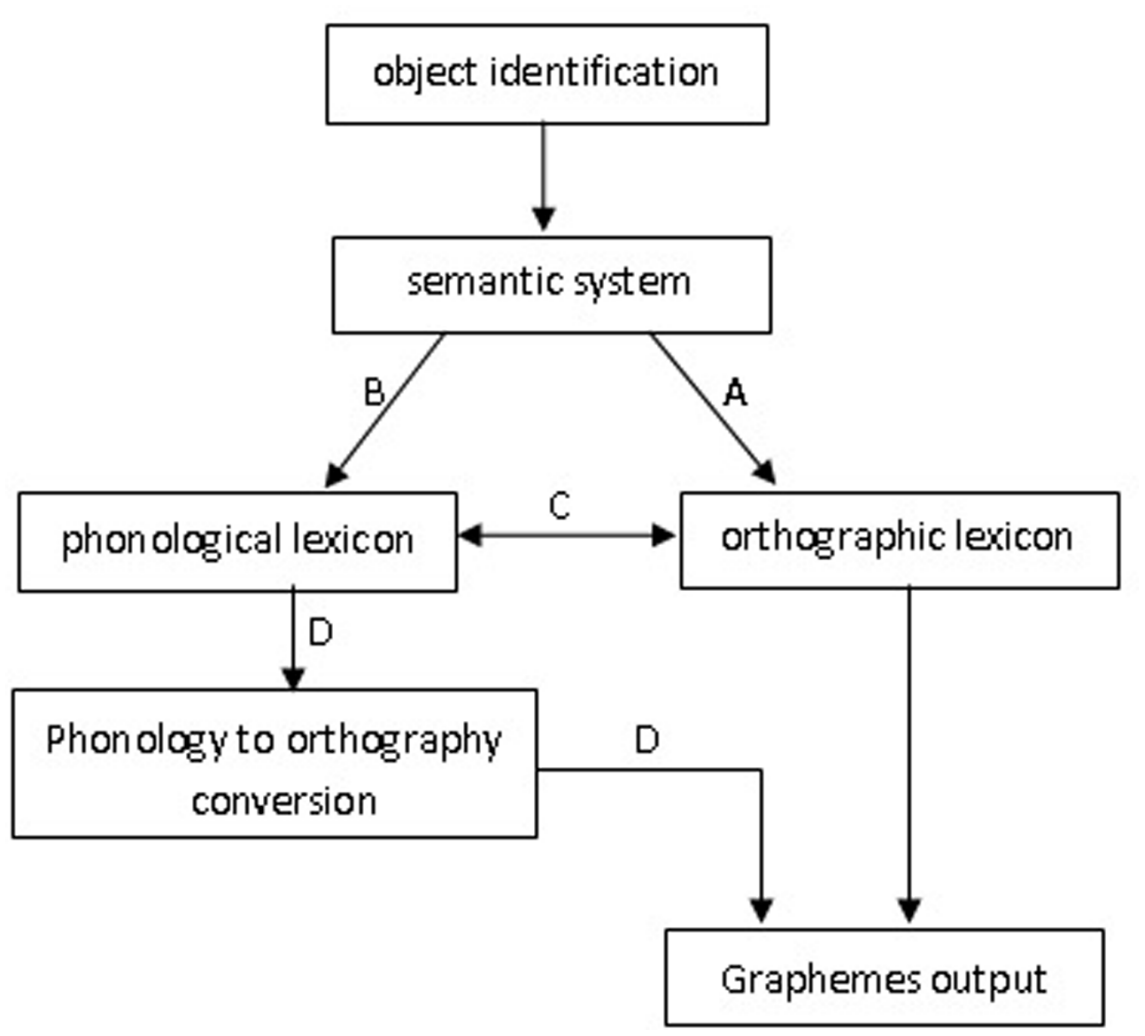
**Sketch model of written picture naming (Bonin et al., 2001)**.

This account, however, does not necessarily imply that intact writing is unaffected by phonological codes in unimpaired individuals. Based on Miceli et al.’s (1997) proposal, Bonin et al. (2001) proposed a working model of written picture naming (see **Figure 1**). When a target picture is presented, the first processing step involves object identification and conceptual preparation in that order. These representations send activation to phonological and orthographic lexicons in parallel, and there are bidirectional connections between two lexicons. The *orthographic autonomy hypothesis* assumes that semantic activation can directly flow to the orthographic lexicon (by link A in **Figure 1**). Miceli et al. (1997) distinguished weak and strong versions of the orthographic autonomy hypothesis. The weak version stipulates that both the orthographic and phonological lexicons are directly activated from the semantic system (by link A and link B in **Figure 1**, respectively) and map directly onto one another (by link C in **Figure 1**). By contrast, the strong version does not acknowledge the links between two lexicons and assumes that phonology may influence orthographic output by a sublexical route (link D). Bonin et al.’s (2001) model suggests that there are lexical (link C) and sublexical (link D) routes from phonology to orthography in written production.

Relatively few empirical studies have addressed the relationship between phonological and orthographic codes with chronometric tasks, and their results have been inconsistent. A few studies demonstrated that phonological codes indeed influence writing (e.g., Bonin et al., 2001; Zhang and Damian, 2010; Afonso and Álvarez, 2011; Damian et al., 2011). Bonin et al. (2001) manipulated the consistency of phonology–orthography mappings at the lexical and sublexical levels in healthy participants to identify the potential effect of phonological codes in written picture naming. The heterographic homophones of picture names carry phonology-to-orthography inconsistencies at both the lexical and sublexical levels. For example, the picture name* cygne* (meaning *swan* in French) and the word *signe* (meaning *sign* in French) are heterographic homophones. The *cygne* was matched with a control picture name, which has no heterographic homophone. Hence, the item in the control condition was consistent, while the target picture name was inconsistent at the lexical level. The inconsistency at the sublexical level was defined at the level of the onset, vocalic and coda units as well as the level of onset plus vowel and vowel plus coda units. Word-initial inconsistencies at the sublexical level were found to affect writing latencies: picture names with inconsistent phono-orthographic mapping (e.g., *oeil* in French) were written more slowly than those with consistent phono-orthographic mapping (e.g., *ongle* in French), whereas no difference was found when consistency was manipulated at the lexical level. This finding further suggests that phonology affects orthographic encoding primarily via a sublexical route.

Zhang and Damian (2010) used a written PWI task to examine the role of phonology in English speakers. Distractors were orthographically and phonologically (OP) related (e.g., picture name: “hand”; distractor: “sand”), orthographically (O) related (e.g., “hand” and “wand”), or unrelated. Zhang and Damian found an exclusive effect of phonology at an stimulus of onset asynchrony (SOA) of 0 ms (OP minus O) and orthographic priming at an SOA of 100 ms, indicating that phonological codes constrain access to orthographic codes at an early stage in written production. By contrast, Bonin et al. (1998b) did not find evidence for the role of phonology in written production in French, which was inconsistent with the findings for English, and this discrepancy casts doubt on the role of phonology in written production.

Evidence for phonological constraints largely stems from studies conducted with alphabetic scripts. This finding is perhaps not surprising for writing systems with alphabetic scripts, in which phonological and orthographic codes are closely interrelated and the relationships between phonology and orthography are quasi-systematic. In non-alphabetic scripts, however, it is less obvious why orthographic retrieval should be affected by phonological codes. Because orthography and phonology are largely dissociated in non-alphabetic scripts, such as Chinese, the orthographic and phonological effects can be appropriately separated from one another in such scripts with an appropriate manipulation. With a PWI task, Qu et al. (2011) manipulated the distractors that were OP related, phonologically (P) related, or unrelated with picture names and with SOAs: 0, 100, and 200 ms. Priming effects were found for both types of related distractors relative to unrelated distracters at 0 and 100 ms SOA, whereas priming from the P related distractors was restricted to 0 ms SOA. These results thus provide evidence that phonological codes are activated rapidly and constrain orthographic output in a non-alphabetic script.

However, there were two potential problems in Qu et al.’s (2011) study. First, the degrees of phonological overlapping are not matched in the OP related and P related conditions. Most Chinese characters contain a so-called “phonetic radical,” i.e., part of a character that indicates how the character as a whole is pronounced. However, the phonetic radical does not always indicate the correct pronunciation of a Chinese character. In Qu et al.’s (2011) study, the phonetic radicals of 15 OP related and 1 P related distractors among 20 distractors can indicate the pronunciation of entire characters [see Qu et al.’s (2011) material sets for details; e.g., picture name: 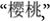 /ying1tao2/, *cherry* in English; the OP distractor: 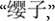 /ying1zi/, *tassel*]. This issue may result in a larger facilitation effect in the OP condition but a smaller facilitation effect in the P condition. Zhao, La Heij and Schiller’s (2012) finding provides evidence for this possibility: a 72-ms phonological facilitation effect at SOA = 0 ms was reduced to 38 ms when phonetic radicals were avoided, reflecting that whether the phonetic radical of a character indicates the pronunciation of the entire character affects the magnitude of the phonological effect. Qu et al. (2011) observed a significant OP effect (31 ms) in the OP condition and a non-significant P effect (15 ms) at SOA = 0 ms, and they inferred that the OP effect is orthographic in nature. Because of the aforementioned confounding factor, it is difficult to conclude that the mixed OP effect originates from orthographically relatedness or phonologically relatedness or from the combination of both. Second, the O related condition was not included in Qu et al.’s (2011) study, and the authors inferred the activation of orthographic codes by comparing the OP effect and the P effect. Our experiment design will avoid these problems.

In the domain of written production, a simple view is proposed on the basis of psycholinguistic and cognitive neuropsychological studies of speech production. Although the spoken and written language production systems may share some processing levels, they also have some specific components (Bonin et al., 1998a). In picture name speaking and writing, a structural level and a semantic level are common, whereas there may be a phonological lexeme level in spoken language but an orthographic lexeme level in handwritten language (Ellis, 1982, 1988; Caramazza and Hillis, 1990; Bonin et al., 1998b). Furthermore, there is a lexical link between phonological and orthographic lexemes (Ellis, 1988; Miceli et al., 1997, 1999; Bonin et al., 1998b). The connection between phonology and orthography is bi-directional: phonological information may serve as input for the articulation process in spoken production, and orthographic information may serve as input for the graphic output process in written production. In the literature on written production, a typical research approach is to compare the processes of written and spoken production to find the dissociation or association between them (i.e., Miceli et al., 1997; Rapp et al., 1997; Bonin et al., 1998a; Damian et al., 2011).

In the present study, we manipulated the OP related, O related, P related, and unrelated distractor words with target names in a PWI task. The SOA interval between the picture and distractor was also varied. The SOAs of -100 ms (distractor onset before pictures), 0 ms, and +100 ms (distractor onset after pictures) were used (Qu et al., 2011). Manipulating the onset of the distractor relative to that of the picture allows us to tap into successive processing stages as a response is being prepared (e.g., Schriefers et al., 1990; Zhang and Damian, 2010; Qu et al., 2011). It is acceptable that negative SOA taps into the early processing stage, while positive SOA taps into the relatively later stage in the PWI task. The participants were asked to write (Experiments 1 and 2) or speak (Experiment 3) the picture names. This approach allows us to directly compare the time course of orthographic and phonological codes in speaking and writing experiments as well as to investigate the contribution of orthographic and phonological codes in written production. We would put emphasis on the relative temporal sequences of orthographic and phonological effects in writing and speaking. Previous studies of Chinese spoken production demonstrated that orthographic and phonological effects are independent (Bi et al., 2009; Zhang and Weekes, 2009; Zhang et al., 2009; Zhao et al., 2012), and the former arises earlier than (Zhang and Weekes, 2009; Zhang et al., 2009) or simultaneously with (Zhao et al., 2012) the latter. Therefore, we predict a time pattern for the O effect and the P effect that is similar to that found for Chinese spoken production.

Concerning the comparison between written and spoken production, according to the *obligatory phonological mediation hypothesis*, written production depends on spoken production; thus, we would expect phonological activation to arise prior to orthographic activation in speaking and writing. According to the *orthographic autonomy hypothesis*, written production is not dependent on spoken production, and orthographic information must be activated in writing and prior to or simultaneously with phonological activation because of the connection between phonological and orthographic lexicons.

## Experiment 1

### Method

#### Participants

Twenty-four students (11 males, average age 23.0 years, age range 19–28 years) participated in the study and were paid approximately $3. All students were native Mandarin Chinese speakers with normal or corrected-to-normal vision.

#### Materials

Fourteen target pictures with monosyllabic names were selected from Zhang and Yang’s (2003) picture database. All pictures had monosyllabic names, with an average lexical frequency of 41.84 per million (Beijing Language Institute, 1986) and an average stroke number of 10. Each picture was paired with three types of form-related monosyllabic distractor words: (1) an orthographically related (O) but phonologically dissimilar word that shared the phonetic radical but no syllable with the picture name [i.e., 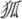 (*fox*, /hu2/) – 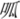 (*crying sound of child*, /gua1/)], (2) a phonologically related (P) but orthographically dissimilar word that shared the syllable but no radical with the picture name [i.e., 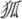 – 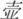 (*pot*, /hu2/)], and (3) an orthographically plus phonologically related word (OP) that shared the phonetic radical and the syllable with the picture name [i.e., 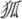 – 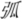 (*arc*, /hu2/)]. For the phonological overlap in the OP and O distractors, both shared the same syllables with target names. For the orthographic overlap in the OP and O distractors, both shared the same phonetic radicals with targets, which are not the first radicals of the target picture names. Importantly, the phonetic radicals of the OP related and P distractor words cannot indicate the pronunciation of the entire characters, and the potential influence of phonetic radicals is excluded (see also Zhao et al., 2012).

The distractors in each condition were then recombined with the picture names to form each corresponding unrelated condition. Semantic or associative relationships between the picture names and distractors were avoided in all combinations. Across three distractor type conditions, the distractor words were statistically matched in the number of strokes and lexical frequency based on normative information reported in the database of the Modern Chinese Frequency Dictionary (Beijing Language Institute, 1986).

#### Design

The experimental design included Relatedness (related vs. unrelated), Distractor Type (O, P, and OP related), and SOA (-100, 0, and 100 ms) as within-participants and within-items factors. For each participant, each picture was displayed under each relatedness and SOA condition, resulting in 342 combinations. Trials were blocked by SOA, and the order in which the participants encountered the SOA blocks varied according to a Latin square design. A new pseudorandom sequence was generated for each participant and each block, with the constraint that neither the targets nor the distractors were repeated on consecutive trials.

#### Apparatus

The experiment was performed using E-Prime Professional Software (Version 1.1). The participants were seated in a quiet room ∼70 cm from a 19-inch LED computer screen. Written responses were recorded with a WACOM Intuos A4 graphic tablet with a WACOM inking digitizer pen.

#### Procedure

The participants were tested individually. They sat in a quiet room at a comfortable viewing distance in front of the computer. The participants were first asked to familiarize themselves with the experimental stimuli by viewing each picture for 3000 ms with the picture name printed below each picture. Then, eight warm-up trials and 114 experimental trials were administered for each SOA block. Each trial involved the following sequence: a fixation point (+) presented in the middle of the screen for 500 ms, followed by a blank screen for 500 ms. Subsequently, the first stimulus (either the distractor or the target) appeared, followed by the appearance of the second stimulus. For SOA = 0 ms, the target and distractor appeared simultaneously.

The distractor words were presented in 25-Song font, centrally superimposed on the target pictures. The pictures were displayed at the bottom of the screen to reduce the participants’ head and eye movements as they wrote the picture names. The participants were asked to write down picture names as quickly and accurately as possible. During the experiment, the participants were instructed to hover the stylus just above the corresponding line on the sheet in anticipation of the response, so that the response would not require an arm movement. An inter-trial interval of 3500 ms concluded each trial. The experiment required ∼40 min in total.

### Results

Data from incorrect responses (1.04%) naming latencies longer than 2000 ms or shorter than 300 ms (1.09%) and those deviating by more than 2.5 SD from the cell mean (1.50%) were removed from all analyses. The remaining data were used in the subsequent statistical analysis. **Table 1** presents the mean latencies and error percentages for Relatedness, Type of Relatedness, and SOA.

**Table 1:** Mean response latencies (RT in milliseconds) and mean error percentages (PE) in Experiment 1.

Condition	SOA					
	-100		0		+100	
	RT	PE	RT	PE	RT	PE
OP_R	957	0.60	913	0.60	923	1.19
OP_U	1007	1.79	961	1.19	967	0.90
Effect	+50^∗∗∗^		+48^∗∗∗^		+44^∗∗∗^	
						
O_R	960	0.89	900	1.19	936	1.19
O_U	1001	0.60	933	0.89	971	1.49
Effect	+41^∗^		+33^∗^		+35^∗^	
P_R	977	1.49	943	0.89	946	1.19
P_U	988	1.19	961	1.19	972	0.30
Effect	+11		+18		+26^∗^	

^∗^*p* < 0.05; ^∗∗∗^*p* < 0.001.

OP = orthographically plus phonologically related; O = orthographically related but phonologically unrelated; P = phonologically related but orthographically unrelated; R = Related; U = unrelated.

We used the lmer program of the lme4 package for estimated fixed effects and parameter estimation of the LMM (Bates, 2005; Baayen et al., 2008). The free software R was used (R Development Core Team, 2009). The data were analyzed using a linear mixed-effects model that included the fixed effects of Relatedness (related vs. unrelated), Distractor Type, and SOA, as well as by-participant and by-item random intercepts. Models were fit to the data using restricted maximum likelihood estimation, which seeks to find the parameter values that make the model’s predicted values most similar to the observed values. Model fitting was performed by initially specifying a model that included only the random factors (participants and items) and was then enriched by subsequently adding the fixed factors Relatedness, Distractor Type and SOA one by one, followed by the interaction between Relatedness and Distractor Type, the interaction between Relatedness and SOA, the interaction of Distractor Type and SOA, and the 3-way interaction among the three fixed variables one by one. The best-fitting model was defined to be the most complex model that significantly improved the fit over the previous model. If adding a fixed factor or an interaction among factors did not significantly improve the fit, then this result indicates that those factors do not significantly influence the dependent variables (i.e., naming latencies).

The results are reported for the best-fitting models. Latencies and errors were analyzed separately for Relatedness, Distractor Type and SOA. For latencies, the best-fitting model included SOA, Relatedness, Distractor Type, and the interaction between Relatedness and Distractor Type. Adding the interaction between SOA and Distractor Type, χ^2^(4,5824) = 3.53, *p* = 0.47, the interaction between SOA and Relatedness, χ^2^(2,5824) = 0.15, *p* = 0.93, and the interaction among three variables, χ^2^(4,5824) = 0.72, *p* = 0.95, did not significantly improve the fit. **Table 2** summarizes the fixed effects of the best model fit.

**Table 2:** Summary of fixed effects in the linear mixed-effects model for onset latencies in Experiment 1.

Effects	Estimate	SE	*t*	*Pr(>|t| )*
(Intercept)	958.47	35.61	26.92	0.0000
SOA: 0 ms	-47.51	5.69	-8.35	0.0000
SOA: 100 ms	-33.25	5.69	-5.84	0.0000
Orthographic relatedness	8.84	8.00	1.10	0.2696
Phonological relatedness	27.04	8.00	3.38	0.0007
Unrelated	52.06	8.05	6.47	0.0000
Unrelated:Orthographically	-25.50	11.38	-2.24	0.0251
Unrelated:Phonologically	-33.15	11.38	-2.91	0.0036

We followed the significant interaction between Relatedness and Distractor Type with separate contrasts. First, we tested for relatedness effects (i.e., unrelated vs. related) separately for each of three types of relatedness. For the OP condition, the results showed that the effects of relatedness differed significantly from one another at -100 ms SOA, *t*(639) = 4.27, *p* < 0.001, 0 ms SOA, *t*(652) = 3.58, *p* < 0.001, and +100 ms SOA, *t*(648) = 4.42, *p* < 0.001. For the O condition, the results showed that the effects of relatedness differed significantly from one another at -100 ms SOA, *t*(647) = 2.11, *p* < 0.05, 0 ms SOA, *t*(647) = 2.38, *p* < 0.05, and +100 ms SOA, *t*(647) = 2.32, *p* < 0.05. By contrast, for the P condition, the results showed that the effects of relatedness differed significantly from one another at 100 ms SOA, *t*(650) = 2.07, *p* < 0.05, but *not* at -100-ms SOA, *t*(646) = 0.98, *p* = 0.32, and 0 ms SOA, *t*(648) = 1.35, *p* = 0.19.

Finally and most importantly, we assessed the degree of additivity between orthographic and phonological relatedness using a formula (see Balota and Paul, 1996; Melinger and Abdel Rahman, 2004 for a similar logic) that indicates the OP effect on the left-hand side (related minus unrelated) and the sum of the O and P effects on the right-hand side.

OP effect = O effect + P effect

If the effects of orthographic and phonological effects are additive, then the two sides of the equation should be statistically equal; if the effects interact, then the two sides of the equation should deviate from zero. Under the assumption that orthographic and phonological variables do not interact, their respective effects should additively summate. For instance, at SOA = -100 ms, **Table 1** shows an orthographic effect of 41 ms (significant) and a phonological effect of 11 ms (insignificant). An additive relationship would predict an effect of 52 ms (41 + 11 ms) for the OP condition. Empirically, the observed OP effect is 50 ms, which is numerically close to the prediction derived from additivity.

We tested this contrast via a coding of [-1, 1, -1, 1, 1, -1] across the orthographically unrelated and related, phonologically related and unrelated, and orthographically plus phonologically unrelated and related cells. This analysis did not return significant results for any of the SOA levels [all *Fs*(1,23) ≤ 0.78, *p* ≥ 0.39], suggesting that orthographic and phonological relatedness did not interact at the three SOAs. Bayesian analysis with the method suggested by Masson (2011) resulted in a Bayes factor of 5.20 with *p*BIC(H_0_|D) = 0.77 and *p*BIC(H_1_|D) = 0.23 at -100 ms SOA, 4.88 with *p*BIC(H_0_|D) = 0.83 and *p*BIC(H_1_|D) = 0.17 at 0 ms SOA, and of 4.90 with *p*BIC(H_0_|D) = 0.83 and *p*BIC(H_1_|D) = 0.17 at +100 ms SOA, which constitutes “positive” evidence for the null hypothesis (i.e., an additive pattern between orthographic and phonological effects) according to the classification suggested by Raftery (1999).

A parallel analysis of variance conducted on the errors showed that none of the models that included SOA, Relatedness, Distractor Type, or two-way or three-way interactions improved the fit, χ^2^ ≤ 2.64, *p*≥ 0.39.

### Discussion

The results showed that the OP related and O related distractors reliably facilitated written production at -100, 0, and +100 ms SOAs, while the P related distractors facilitated written production at +100 ms SOA. Critically, the difference between the OP related effect and the O related effect was not significant at -100 ms SOA. At -100 ms SOA, the magnitude of the OP effect (50 ms) was significantly larger than that of the P effect (11 ms) but was not larger than the magnitude of the O effect (41 ms), reflecting that the OP effect was primarily orthographic, not phonological. Furthermore, the formula analysis indicates that the O effect and the P effect did not interact but rather exerted an additive effect. The present finding shows that phonology is not accessed earlier than orthography in Chinese written production. By contrast, Qu et al. (2011) found that phonology is activated before orthography (see also Zhang and Damian, 2010). Therefore, our finding is not consistent with previous data. To examine the reliability of our findings, we aim to replicate the findings of Experiment 1 in Experiment 2.

## Experiment 2

### Method

#### Participants

Twenty-four students (10 males, average age 22.2 years, age range 19–26 years) from the same pool participated in the experiment and were paid approximately $6.

#### Materials

Twenty-five black-and-white pictures with monosyllabic names were selected, including 14 target pictures and 11 fillers. There were five semantic categories (animals, tools, housewares, weapons, and music instruments), and each category consisted of five pictures. We deleted three pictures used in experiment 1 (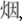 cigarette, /yan1/; 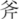, ox, /fu3/, and 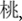 peach, /tao2/) because of the phoneme overlap between phonetic radicals and entire character pronunciation, and we added three other target pictures (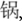 pot, /guo1/, 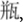 bottle, /ping2/, and 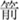, arrow, /jian4/). Similar to Experiment 1, each target picture was paired with three types of distractor words (OP, O, and P) and then recombined to form corresponding unrelated conditions. Each filler picture was also paired with three unrelated distracter words, and the distractors in each condition were then recombined with the picture names to form other unrelated conditions.

The design, apparatus, procedures, and other aspects of the materials were identical to those used in Experiment 1.

### Results

Data for target pictures were analyzed. Incorrect responses and other responses such as “well” or hesitations (0.79%), naming latencies longer than 2000 ms or shorter than 300 ms (1.04%), and those deviating by more than 2.5 SD from the cell means (2.60%) were removed from all analyses. The remaining data were used in the subsequent statistical analysis. **Table 3** presents the mean latencies and error percentages for Relatedness, Distractor Type, and SOA.

**Table 3:** Mean response latencies (RT in milliseconds) and mean error percentages (PE) in Experiment 2 (^∗^*p* < 0.05; ^ł^*p* < 0.10; ^∗∗∗^*p* < 0.001).

Condition	SOA					
	-100		0		+100	
	RT	PE	RT	PE	RT	PE
OP_R	863	0	850	0	834	0.30
OP_U	897	0.60	898	0.89	873	0.60
Effect	+34^∗∗∗^		+48^∗∗∗^		+39^∗∗∗^	
						
O_R	870	0	864	0	836	0.60
O_U	896	0.89	893	0.60	874	0.60
Effect	+26^∗^		+29^∗∗∗^		+38^∗∗∗^	
						
P_R	875	0	860	0	868	0.60
P_U	886	0.89	881	0.30	877	0.60
Effect	+11		+21^ł^		+9	

The results are reported for the best-fitting models. Latencies and errors were analyzed separately for Relatedness, Distractor Type, and SOA. For latencies, the best-fitting model included SOA, Relatedness, and the interaction between Relatedness and Distractor Type. Adding Distractor Type, χ^2^(2,5775) = 1.78, *p* = 0.40, the interaction between SOA and Distractor Type, χ^2^(4,5775) = 5.35, *p* = 0.25, the interaction between SOA and Relatedness, χ^2^(2,5775) = 1.81, *p* = 0.40, and the interaction among three variables, χ^2^(4,5775) = 0.22, *p* = 0.99, did not significantly improve the fit. **Table 4** summarizes the fixed effects of the best-fitting model.

**Table 4:** Summary of fixed effects in the linear mixed-effects model for onset latencies in Experiment 2.

Effects	Estimate	SE	*t*	*Pr(>|t| )*
(Intercept)	862.74	35.15	24.54	0.0000
SOA: 0 ms	-3.80	4.56	-0.83	0.4048
SOA: 100 ms	-15.37	4.57	-3.37	0.0008
Unrelated	42.58	6.45	6.60	0.0000
Unrelated:Orthographically	-0.03	6.49	-0.01	0.1788
Unrelated:Phonologically	-8.51	6.47	-1.32	0.9960
Related:Orthographically	8.64	6.43	1.35	0.0022
Related:Phonologically	19.74	6.43	3.07	0.1881

A follow-up analysis similar to that used in Experiment 1 was conducted. For the OP condition, the results showed that the effects of relatedness differed significantly from one another at -100 ms SOA, *t*(653) = 3.75, *p* < .001, 0 ms SOA, *t*(640) = 4.34, *p* < 0.001, and +100 ms SOA, *t*(634) = 4.51, *p* < 0.001. For the O condition, the results showed that the effects of relatedness differed significantly from one another at -100 ms SOA, *t*(647) = 2.34, *p* < 0.05, 0 ms SOA, *t*(639) = 3.65, *p* < 0.001, and +100 ms SOA, *t*(633) = 3.92, *p* < 0.001. For the P condition, the results showed that the effects of relatedness did not differ significantly from one another at 0 ms SOA, *t*(642) = 0.98, *p* = 0.33, 0 ms SOA, *t*(644) = 1.70, *p* = 0.09, and +100 ms SOA, *t*(643) = 1.30, *p* = 0.19.

We again tested the contrasting effects via a coding of [-1, 1, -1, 1, 1, -1] across the orthographically unrelated and related, phonologically related and unrelated, and orthographically plus phonologically unrelated and related cells. This analysis did not return significant results for any SOA levels [all *Fs*(1,23) ≤ 0.12, *p* ≥ 0.49], suggesting that orthographic and phonological relatedness did not interact. Bayesian analysis as performed in Experiment 1 resulted in a Bayes factor of 4.52 with *p*BIC(H_0_|D) = 0.82 and *p*BIC(H_1_|D) = 0.18 at -100-ms SOA, of 4.10 with *p*BIC(H_0_|D) = 0.80 and *p*BIC(H_1_|D) = 0.20 at 0-ms SOA, and of 3.76 with *p*BIC(H_0_|D) = 0.79 and *p*BIC(H_1_|D) = 0.21 at +100-ms SOA, which constitutes “positive” evidence for the null hypothesis of additive effects of orthographic and phonological relatedness (Raftery, 1999).

A parallel analysis of variance conducted on the errors showed that none of the models that included SOA, Relatedness, Distractor Type, or two-way or three-way interactions improved the fit, χ^2^ ≤ 4.47, *p*≥ 0.11.

### Discussion

The results showed that the OP related and O related distractors reliably facilitated written production at -100, 0, and +100 ms SOAs, and these findings replicated the results of Experiment 1, while the P related distractors produced a marginally significant facilitation effect at 0 ms SOA. As in the first experiment, the difference between the OP related effect and the O related effect was not significant, whereas the difference between the OP effect and the P effect was significant, indicating that the OP effect was primarily orthographic, not phonological. Statistical tests on this formula also indicated that orthographic relatedness and phonological relatedness did not interact, which is perfectly consistent with the findings in Experiment 1. Experiments 1 and 2 hence provide converging evidence for an additive pattern of orthographic and phonological relatedness and indicate that phonology is not accessed earlier than orthography in Chinese written production.

There were some important divergences in Experiments 1 and 2. The magnitude of the OP effect and the O effect were comparable in both experiments. By contrast, there were significant phonological effects (26 ms) at +100 ms in Experiment 1 versus a marginally significant effect (21 ms) at 0 ms in Experiment 2. The absence of the P effect may result from the relatively larger picture sets: there were 14 target pictures in the first experiment and 25 in the second experiment. In both experiments, each target picture was presented and named 18 times by each participant, which is a common manipulation in studies using the PWI paradigm. The classic semantic interference and form-related facilitation cannot be eliminated by repetition in spoken responses, so it is usually acceptable in studies and not perceived as problematic. However, Roelofs (2001) proposed that when target names are repeated many times, causing participants to establish a response set in memory, even the number of target names slightly exceeds the short-term memory span, which may include 12–16 responses. Therefore, it was easier to establish a response set for 14 items in Experiment 1 than for 25 items in Experiment 2. However, the percentage of phonologically related distracters was 16.7% in Experiment 1 compared with 9.33% in Experiment 2. Therefore, it could be argued that the results of Experiment 2 underestimate the effects of phonology in handwriting for those two reasons. Although we acknowledge this possibility, it should be noted that the situations of OP related and O related conditions were identical to the P related condition, but the OP effect and the O effect were comparable in both experiments; only the P effect was absent in Experiment 2. Therefore, our results indicate that the phonological facilitation effect was vulnerable to the number of picture sets and the percentage of phonologically related trials in total, while the orthographic facilitation effect was stable and reliable. Hence, the influence of phonological codes in handwritten production may not be mandatory and universal.

## Experiment 3

In the literature, cognitive neuropsychological studies have reported an experimental dissociation in writing and speaking (Miceli et al., 1997; Rapp et al., 1997). Researchers have also proposed written production models on the basis of speech production. Therefore, we conducted a speaking experiment using the same stimuli and experimental paradigm. This approach allows us to compare the time course of orthographic and phonological codes in speaking and writing in normal adults. As mentioned in the introduction, such a comparison would provide relevant evidence for the argument of the phonological mediation hypothesis and the orthography autonomy hypothesis.

### Method

#### Participants

Six months after Experiment 1 was conducted, the same 22 participants in Experiment 1 returned and participated in Experiment 3, and they reported that they had little memory of Experiment 1. Two more participants were recruited from the same pool of participants. All participants were paid approximately $4.

The *materials, design, apparatus, and procedure* were identical to those used in Experiment 1, except that the participants were asked to name the target aloud as quickly and accurately as possible, and naming latencies were measured from target onset using a voice-key connected with the computer via a PST Serial Response Box. The inter-trial interval was 1500 ms, and the experiment took ∼30 min in total.

### Results

Data from incorrect responses (1.49%), naming latencies longer than 1500 ms or shorter than 200 ms (1.39%), and those deviating by more than 2.5 SD from the cell mean (2.96%) were removed from all analyses. The remaining data were used in the subsequent statistical analysis. **Table 5** presents the mean latencies and error percentages for Relatedness, Distractor Type, and SOA.

**Table 5:** The mean latencies and error percentages for Relatedness, Distractor Type, and SOA in Experiment 3.

Condition	SOA					
	-100		0		+100	
	RT	PE	RT	PE	RT	PE
OP_R	622	0.60	601	0	629	1.19
OP_U	695	0.89	698	1.79	681	2.38
Effect	+73^∗∗∗∗^		+97^∗∗∗∗^		+52^∗∗∗∗^	
O_R	643	0.30	613	0.89	632	0.89
O_U	695	0.60	680	0.19	667	0.89
Effect	+52^∗∗∗∗^		+67^∗∗∗∗^		+35^∗∗∗∗^	
P_R	650	0.30	631	0.60	630	0.30
P_U	678	1.79	685	2.38	680	2.38
Effect	+28^∗∗∗∗^		+54^∗∗∗∗^		+50^∗∗∗∗^	

^∗∗∗∗^*p* < .0001.

The results are reported for the best-fitting models. Latencies and errors were analyzed separately. For latencies, the best-fitting model included SOA, Relatedness, the interaction between SOA and Distractor Type, the interaction between Relatedness and Distractor Type, the interaction between Relatedness and Distractor Type, and the three-way interaction. Adding Distractor Type did not significantly improve the fit, the interaction between SOA and Distractor Type, χ^2^(2,5720) = 2.88, *p* = 0.24. **Table 6** summarizes the fixed effects of the best-fitting model fit.

**Table 6:** Summary of fixed effects in the linear mixed-effects model for onset latencies in Experiment 3.

Effects	Estimate	SE	*t*	*Pr(>|t| )*
(Intercept)	700.15	18.84	37.15	0.0000
SOA2	2.97	7.59	0.39	0.6959
SOA3	-10.946	7.62	-1.44	0.1508
related2	-75.28	7.502	-10.04	0.0000
SOA1:type2	-0.408	7.55	-0.05	0.9572
SOA2:type2	-20.97	7.61	-2.75	0.0060
SOA3:type2	-20.218	7.57	-2.67	0.0076
SOA1:type3	-17.06	7.62	-2.24	0.0251
SOA2:type3	-12.74	7.61	-1.67	0.0944
SOA3:type3	-4.364	7.63	-0.57	0.5673
SOA2:related2	-25.85	10.60	-2.44	0.0148
SOA3:related2	17.82	10.66	1.67	0.0946
related2:type2	21.40	10.60	2.02	0.0434
related2:type3	44.39	10.64	4.17	0.0000
SOA2:related2:type2	11.41	15.00	0.76	0.4468
SOA3:related2:type2	2.55	15.01	0.17	0.8650
SOA2:related2:type3	-0.48	15.03	-0.03	0.9745
SOA3:related2:type3	-39.12	15.07	-2.60	0.0095

SOA1: -100 ms; SOA2: 0 ms; SOA 3: 100 ms; type2: orthographic relatedness; type3: phonological relatedness; related2: unrelated.

An analysis similar to that performed in Experiment 1 was conducted. For the OP condition, the results showed that the effects of relatedness differed significantly from one another at -100 ms SOA, *t*(638) = 10.34, *p* < 0.0001, 0 ms SOA, *t*(641) = 13.98, *p* < 0.0001, and +100 ms SOA, *t*(626) = 7.10, *p* < 0.0001. For the O condition, the results showed that the effects of relatedness differed significantly from one another at -100 ms SOA, *t*(641) = 7.32, *p* < 0.0001, 0 ms SOA, *t*(633) = 9.02, *p* < 0.0001, and +100 ms SOA, *t*(643) = 4.95, *p* < 0.0001. For the P condition, the results showed that the effects of relatedness differed significantly from one another at -100 ms SOA, *t*(632) = 4.42, *p* < 0.0001, 0 ms SOA, *t*(632) = 7.67, *p* < 0.0001 and +100 ms SOA, *t*(634) = 6.93, *p* < 0.0001.

We again tested the contrasting effects via a coding of [-1, 1, -1, 1, 1, -1] across the orthographically unrelated and related, phonologically related and unrelated, and orthographically plus phonological unrelated and related cells. This analysis did not return significant results at -100 ms SOA, *F*(1,23) = 0.900, *p* = 0.353, but they were significant at 0 ms SOA, *F*(1,23) = 4.611, *p* = 0.043 and marginally significant at 100 ms SOA, *F*(1,23) = 3.374, *p* = 0.079, suggesting that orthographic and phonological relatedness did not interact at an earlier stage but did interact at a later stage. Bayesian analysis as conducted in Experiment 1 resulted in a Bayes factor of 3.09 with *p*BIC(H_0_|D) = 0.76 and *p*BIC(H_1_|D) = 0.24 at -100 ms SOA, which constitutes “positive” evidence for the null hypothesis of additive orthographic and phonological effects according to Raftery (1999). By contrast, the Bayes factor was 0.55 with *p*BIC(H_0_|D) = 0.35 and *p*BIC(H_1_|D) = 0.65 at 0 ms SOA, and 0.95 with *p*BIC(H_0_|D) = 0.49 and *p*BIC(H_1_|D) = 0.51 at +100 ms SOA, which constitutes “weak” evidence for the interaction between orthographic and phonological relatedness in spoken production (Raftery, 1999).

A parallel analysis of variance was conducted on the errors, but binomial data were used. The best-fitting model included Relatedness and the interaction between Relatedness and Distractor Type. Adding SOA, Distractor Type, the interaction between SOA and Distractor Type, the interaction between SOA and Relatedness, and the three-way interaction among three variables did not improve the fit, χ^2^ ≤ 3.07, *p*≥ 0.31. A planned comparison showed a significant difference in the error rate in the P condition, -100 ms, *z* = 1.81, *p* = 0.07; 0 ms, *z* = 2.23, *p* = 0.03; 100 ms, *z* = 0.69, *p* = 0.49. Other comparisons were not significant, *zs* ≤ 1.55, *p*≥ 0.12.

#### Combination Analysis of Written and Spoken Production

A total of 22 individuals participated in both Experiments 1 and 3; thus, we performed a combination analysis for onset latencies in writing and speaking. A factor of output modality (written and spoken production) was added to the previous model (SOA, Distractor Type, and Relatedness). The best-fitting model included SOA; Relatedness; Modality; the interaction between SOA and modality; the interaction between relatedness and modality; and the three-way interaction among Distractor Type, Relatedness, and Modality. The model fit did not significantly improve by adding Distractor Type, χ^2^(2,10487) = 2.61, *p* = 0.27; the interaction between SOA and Distractor Type, χ^2^(4,10487) = 2.93, *p* = 0.57; the interaction between SOA and Relatedness, χ^2^(2,10487) = 3.52, *p* = 0.17; the interaction between Relatedness and Distractor Type, χ^2^(2,10487) = 0.44, *p* = 0.80; the interaction between Distractor Type and Modality, χ^2^(2,10487) = 0.56, *p* = 0.76; the triple interaction among SOA, Distractor Type and Relatedness, χ^2^(4,10487) = 1.77, *p* = 0.78; the triple interaction among SOA, Distractor Type and Modality, χ^2^(4, 10487) = 1.90, *p* = 0.75; the triple interaction among SOA, Relatedness and Modality, χ^2^(2,10487) = 2.87, *p* = 0.24; and the 4-way interaction among four factors, χ^2^(4,10487) = 2.86, *p* = 0.58.

### Discussion

The results showed that the OP related, O related and P related distractors reliably facilitated spoken production at -100, 0, and +100 ms SOAs. The striking findings were that the P effect occurred simultaneously with the O effect (see Zhao et al., 2012 for a similar finding), and the formula analysis indicates that the O effect and the P effect did not interact at -100 ms SOA, although they did interact at 0 and 100 ms SOAs. Our findings are consistent with previous studies (Bi et al., 2009; Zhang and Weekes, 2009; Zhang et al., 2009). At an earlier stage of spoken production, we found a similar additive pattern of orthography and phonology in Chinese (Zhang et al., 2009), reflecting that the role of orthography is independent of phonology, and the orthographic distractor may activate its semantic representation at the conceptual level, thereby facilitating the conceptual identification of the target picture. At a later stage of spoken production, we found an interaction between orthographic and phonological effects, reflecting that the orthographic distractor may directly activate the corresponding phonological representation and facilitate the production of a vocal response.

## General Discussion

In an adaptation of the PWI task, we investigated the temporal courses of orthographic and phonological activation in spoken and written production and addressed the contribution of orthography and phonology to written production. The results showed that the OP related and O related distractors reliably facilitated written and spoken production at -100, 0, and +100 ms SOAs. Critically, the P related effect was significant at a later stage of written production, and the difference between the OP and O conditions was not significant at -100 ms SOA in written production. By contrast, the P related effect was significant at an earlier stage and persisted in the later stage of spoken production. Based on the assumption that the manipulation of SOA in PWI studies allows insight into the picture-naming and picture-writing process as it unfolds over time (Qu et al., 2011), our findings at least suggest that phonology is not accessed earlier than orthography in written production, although it is accessed simultaneously with orthography in spoken production. We therefore suggest that the output modality influences the temporal pattern of phonological effects. Additionally, orthographic and phonological effects are additive in written production. However, the two effects are additive and then interact in spoken production. The findings provide evidence that orthography is not mediated by phonological information, in accordance with the orthographic autonomy hypothesis (Miceli et al., 1997; Rapp and Caramazza, 1997; Bonin et al., 1998b).

The present findings on written production are not consistent with the conclusions for English and Chinese within the PWI paradigm. In previous studies, Zhang and Damian (2010) for English and Qu et al. (2011) for Chinese found that phonology is accessed earlier than orthography. How can we interpret the reverse pattern found between studies? Our study differed from previous works in critical ways. First, previous studies inferred the temporal courses of orthography and phonology indirectly. Zhang and Damian (2010) used OP related and O related conditions in English, and they inferred the P effect by comparing the OP effect and the O effect. Qu et al. (2011) inferred the O effect by comparing the OP effect and the P effect. If we used only the OP and P conditions in the present study as did Qu et al. (2011), because of the lack of significant difference between the OP effect and the P effect at 0 and 100 ms SOAs in Experiment 1, we would come to the same conclusion: phonology is accessed earlier than orthography in written Chinese. However, importantly, when the OP, O, and P conditions were introduced into the written production system simultaneously, we observed patterns that were distinct from Qu et al.’s (2011) study with respect to the temporal course of orthographic information. The second difference was that we considered -100 ms SOA in the present study. Compared to positive SOAs, the negative SOA (i.e., -100 ms) taps into the early processing stage of written production. At -100 ms SOA, the magnitude of the OP effect (50 ms in Experiment 1 and 34 ms in Experiment 2) was significantly larger than that of the P effect (11 ms in Experiments 1 and 2) but was not larger than the O effect (41 ms in Experiment 1 and 26 ms in Experiment 2); hence, the OP effect was primarily orthographic, not phonological. Qu et al. (2011) demonstrated that the observed phonological facilitation effect could not be attributed to priming by feedback from the phonological to conceptual level. Therefore, our findings provide consistent and reliable evidence that orthographic codes are accessed rapidly at an earlier stage, whereas phonological codes are accessed at a later stage in writing. According to the obligatory phonological mediation hypothesis, phonological activation should be activated before or simultaneously with orthographic activation in written production. Our findings therefore provide evidence for the orthographic autonomy hypothesis rather than the obligatory phonological mediation hypothesis.

As summarized in the introduction, the evidence with regard to whether handwriting is constrained by phonological codes was from healthy individuals (Bonin et al., 2001; Zhang and Damian, 2010) and patients (e.g., Miceli et al., 1999; Folk and Jones, 2004). Bonin et al. (2001) found an initial implication of phonological codes in written picture naming by varying the sound-to-print consistency of picture labels and the position of inconsistent units, suggesting that the build-up of orthographic activation from pictures is phonologically constrained via a route of sublexical conversion. Miceli et al. (1999) reported a patient ECA who produced inconsistent responses in the say-then-write condition but not in the write-then-say condition. The patient ECA was tested, and it was found that he had damage to the semantic system and to sublexical phoneme–grapheme conversion but not to sublexical grapheme-to-phoneme conversion. Hence, ECA generated inconsistent responses in only one direction in the double naming task, and this pattern contrasts with those that produce inconsistent lexical responses either in both say-then-write and write-then-say conditions or in neither condition. Those researchers therefore propose that phonological and orthographic lexical forms can be accessed autonomously but that they interact through sublexical conversion processes. By contrast, the present finding of early orthographic activation indicates that orthographic codes can be accessed without phonological mediation. A possibility for the divergence between studies was that different target languages were used in the work of Bonin et al. (2001) (French) and our study (Chinese). Orthography and phonology are related in alphabetic scripts such as English or French, whereas they are largely dissociated in non-alphabetic scripts such as Chinese. Therefore, it is perhaps not surprising to find an influence of phonology on orthographic output in the case of alphabetic languages.

However, findings on the role of phonology in written production have not been consistent for alphabetic languages (Bonin et al., 1998b, 2001, 2014; Roux and Bonin, 2011). For example, Bonin et al. (1998b) employed a masked priming task in which participants produced the written names of pictures and in which word primes were presented for a short duration before the pictures and were forward and backward masked. The researchers found an orthographic facilitation effect when primes were presented for 34 and 51 ms, which, crucially, was not modulated by homophonic primes (*pype-pipe*) or non-homophonic primes (*pope-pipe*). This finding was used to argue against the role of phonology in writing. Furthermore, Roux and Bonin (2011) did not find a phonological facilitation effect using a picture–picture interference paradigm, and they suggested that phonological codes are less strongly involved in orthographic encoding. Recently, Bonin et al. (2014) found that the involvement of lexical and sublexical levels depends on the different types of written production tasks. These contradicting results for the same language question the role of phonology in handwritten production, even in alphabetic scripts. Further studies are needed to investigate the divergence among different script systems.

The different temporal patterns and interaction of orthographic and phonological effects in written and spoken production suggest that the output modality may influence the interplay of orthography and phonology in language production. Orthographic and phonological effects are additive in general, and both effects can occur independently in written production. By contrast, the two effects are additive at an earlier stage but interact at a later stage of spoken production (see Zhang et al., 2009 for similar findings but with a slightly different experimental design). If written production depends on spoken production, then we should find a similar temporal pattern of phonological and orthographic effects in written to spoken production: a phonological effect arises simultaneously with an orthographic effect. Therefore, this finding also indicates that written production is not dependent on spoken production and is thus consistent with the argument of the orthographic autonomy hypothesis.

In parallel to the issue of a phonological role in written production, an issue in spoken production is the role of orthography. Recent findings further suggest that orthography plays a role only when it is relevant to a spoken word production task (Roelofs, 2006; Schiller, 2007; Bi et al., 2009; Zhang and Damian, 2010). In our study, the orthographic effect was observed regardless of the output modality. This finding may result from the visual distractors in the PWI task. Damian and Bowers (2009) found that the orthographic effect disappeared when distractors are presented auditorily in a PWI task. These findings indicate that the interplay between orthography and phonology is weak in language production (both spoken and written) in Chinese. Note that although contrast analysis revealed a significant interaction between orthographic and phonological relatedness in spoken production, the Bayes factor with *p*BIC(H_0_|D) and *p*BIC(H_1_|D) constitutes only “weak” evidence for the interaction between them.

What are the implications of our findings for written production? The written model proposed by Bonin et al. (2001) assumes a semantic system that is symmetrically linked to both a phonological and orthographic output lexicon. Both lexicons also directly map onto one another (link C in **Figure 1**), implying that the selection of a graphemic entry is influenced by both direct activation from the semantic system (link A in **Figure 1**) and indirect activation from the phonological lexicon via a lexical route (links B and C in **Figure 1**). Within such a framework, our finding of an early processing stage in which priming is dominated by orthographic relatedness suggests that the activation occurs quickly and directly from the semantic system to graphemic codes. A relatively late phonological effect suggests that activation could occur via the phonological pathway and then be transmitted to the graphemic lexicon indirectly. In the P related condition, targets and distractors do not share any sublexical components except the phonological syllable. Syllables would map onto characters consisting of target names, and in comparison with an unrelated condition, the P related distractors would generate a facilitation effect. We therefore suggest that orthographic codes can be accessed via a lexical route rather than a sublexical route in the present study. We do not deny the role of phonology in written production, but phonological influence was not mandatory and universal, and it can be modulated via experimental manipulations (see Zhang and Damian, 2010 for a similar conclusion based on an articulation suppression task).

## Conclusion

We found an orthographic effect at an early stage in written production, reflecting a fast and direct link between meaning and graphemic lexicon, and we demonstrated that orthographic codes can be accessed directly from meaning in healthy adults. We also found orthographic and phonological effects at a later stage, reflecting a slow and indirect link between meaning and graphemic lexicon via phonology. Furthermore, the absence of an interaction effect of orthographic and phonological facilitation on written latencies suggests that the two effects are additive in general but that they might occur independently in written production in Chinese. Concerning the process of spoken production, we found that orthographic and phonological effects occur simultaneously and that both effects are additive at an early stage but interactive at a later stage. The temporal courses and their interplay of orthographic and phonological effects are dissociative in written and spoken production. Our findings support the orthography autonomy hypothesis, rather than obligatory phonological mediation hypothesis, for written production.

## Conflict of Interest Statement

The authors declare that the research was conducted in the absence of any commercial or financial relationships that could be construed as a potential conflict of interest.
